# Identification of new diagnostic biomarkers for *Mycobacterium tuberculosis* and the potential application in the serodiagnosis of human tuberculosis

**DOI:** 10.1111/1751-7915.13291

**Published:** 2018-06-27

**Authors:** Ningning Ren, Jingfang JinLi, Yingyu Chen, Xia Zhou, Jieru Wang, Pan Ge, Farhan Anwar Khan, Li Zhang, Changmin Hu, Ian D. Robertson, Huanchun Chen, Aizhen Guo

**Affiliations:** ^1^ The State Key Laboratory of Agricultural Microbiology Wuhan 430070 China; ^2^ College of Veterinary Medicine Huazhong Agricultural University Wuhan 430070 China; ^3^ Tuberculosis Department Wuhan Medical Treatment Center Wuhan 430023 China; ^4^ Department of Animal Health Faculty of Animal Husbandry and Veterinary Sciences The University of Agriculture Peshawar Khyber Pakhtunkhwa 25120 Pakistan; ^5^ Hubei International Scientific and Technological Cooperation Base of Veterinary Epidemiology Huazhong Agricultural University Wuhan 430070 China; ^6^ College of Veterinary Medicine Murdoch University Murdoch WA 6160 Australia

## Abstract

*Mycobacterium tuberculosis* (*M. tuberculosis*) regions of difference (RD) encode proteins which are potentially useful as diagnostic reagents for tuberculosis (TB). In this study, 75 genes from *M. tuberculosis* RD1‐RD16 were successfully cloned from which 68 proteins were expressed and purified. Three serum pools from patients with pulmonary TB (PTB), extra‐pulmonary tuberculosis (EPTB) and healthy controls (HC) were used to preliminarily screen individual RD proteins. The OD_630_ ratio of the PTB or EPTB to the HC group ≥ 2‐fold was positive. As a result, 29 proteins were obtained. The serological response to the identified antigens was further verified using 58 PTB samples with 38 sera from smear‐positive PTB (PTB‐SP) patients and 20 sera from smear‐negative PTB (PTB‐SN) patients, 16 EPTB samples, 42 latent *M. tuberculosis* infection samples and 40 HCs by indirect ELISA. With respect to the PTB diagnosis, receiver operating characteristic analysis showed that Rv0222 [area under the curve (AUC), 0.8129; 95% confidence interval (CI), 0.7280–0.8979] and Rv3403c (AUC, 0.8537; 95% CI, 0.7779–0.9294) performed better than ESAT6/CFP10 (AUC, 0.7435; 95% CI, 0.6465–0.8406). Rv0222 and Rv3403c demonstrated the highest diagnostic ability in the PTB‐SP group (sensitivity, 86.8%; specificity, 80%), while Rv3403c demonstrated the highest diagnostic ability in the PTB‐SN group (sensitivity, 70%; specificity, 80%). With respect to the EPTB diagnosis, Rv0222 exhibited the highest diagnostic value (AUC, 0.7523; sensitivity, 68.8%; specificity, 87.5%). In addition, the combination of Rv0222 and Rv3403c improved the test for PTB‐SN. These results indicate that Rv0222 and Rv3403c would be potential diagnostic biomarkers for active TB serodiagnosis. Mouse experiments demonstrated that Rv0222 and Rv3403c elicited specific cellular and humoral responses which were characterized by production of IFN‐γ, IgG1, and IgG2a, but a higher level of IgG1 than IgG2a.

## Introduction

Tuberculosis (TB), caused by *Mycobacterium tuberculosis* (*M. tuberculosis*), has a high rate of infection and death and is one of the most serious global infectious diseases (You *et al*., 2017). In 2016, the World Health Organization (WHO) estimated there were 10.4 million new cases and 1.3 million deaths resulting from TB. China is among the 30 countries with a high burden of TB, as reported by the incidence and mortality of 895 000 and 50 000 cases, respectively (WHO, 2017). In addition, one‐third of the global population is estimated to be infected by *M. tuberculosis* in a latent stage that could potentially turn into active TB at any time. Recently, the WHO created ‘The END TB Strategy’ (2016‐2035), which has a goal of ending TB in humans by 2035 (Uplekar *et al*., 2015). It is well‐known that a sensitive, specific, rapid and simple diagnosis is imperative for the timely and effective treatment of TB, especially for smear‐negative pulmonary TB (PTB‐SN) patients (Alavi‐Naini *et al*., 2012). Therefore, the diagnostic technique is critical in implementing the aforementioned WHO strategy (Sweeney *et al*., 2016).

The current techniques used in diagnosing TB have several disadvantages. For example, sputum smear microscopy is fast and inexpensive, but is of low sensitivity and specificity. Mycobacterial isolation is regarded as the gold standard, but is time‐consuming and has low sensitivity (Xiao *et al*., 2017). In addition, mycobacterial isolation requires a high level of biosafety facilities. Similarly, a diagnosis based on chest images is of low sensitivity and specificity and often has a high cost. The tuberculin skin test (TST) is highly sensitive, but has low specificity, which cannot distinguish Bacille Calmette‐Guerin (BCG) vaccination from natural infection (Pai *et al*., 2014). The IFN‐γ *in vitro* release assay (IGRA) has improved sensitivity or specificity; however, the IGRA lacks consistency and reproducibility (Herrera *et al*., 2011). The Xpert^®^ MTB/RIF assay (Cepheid, Sunnyvale, CA, USA) can detect both TB and rifampicin resistance within 2 h, but its higher cost makes it difficult to be popularized worldwide, especially in underdeveloped and developing countries (Trebucq *et al*., 2011; Steingart *et al*., 2014).

Therefore, TB diagnostic methods require further improvement. The seroreactive protein biomarkers used for TB diagnosis are of priority to be considered due to easy detection. In addition, diagnosis results are retrospective, and the test methods can be simplified and automated. Some of the *M. tuberculosis* proteins encoded by regions of difference (RDs) are potential candidate biomarkers capable of differentiating *M. tuberculosis* infection from BCG vaccination (Chen *et al*., 2009; Al‐Khodari *et al*., 2011). The comparative genome analysis revealed that there are 16 RDs including 129 differential open reading frames (ORFs) between *M. tuberculosis* and BCG (Behr *et al*., 1999); however, most of these identified diagnostic markers show high sensitivity in smear‐positive pulmonary TB(PTB‐SP) patients, but low sensitivity in PTB‐SN and extra‐pulmonary tuberculosis (EPTB) cases (Abebe *et al*., 2007; Beyene *et al*., 2010). Therefore, efforts are still needed to screen novel and more effective markers for TB diagnosis, especially for PTB‐SN and EPTB cases.

This study aimed to clone and express all 129 ORFs within the 16 RDs of *M. tuberculosis* and screen the seroreactive protein markers individually with serum samples from different types of TB patients and healthy controls (HCs). The most specific and sensitive *M. tuberculosis* proteins were further used to establish a novel TB diagnostic assay with improved accuracy.

## Results

### Generation of RD antigens

**Figure 1:**
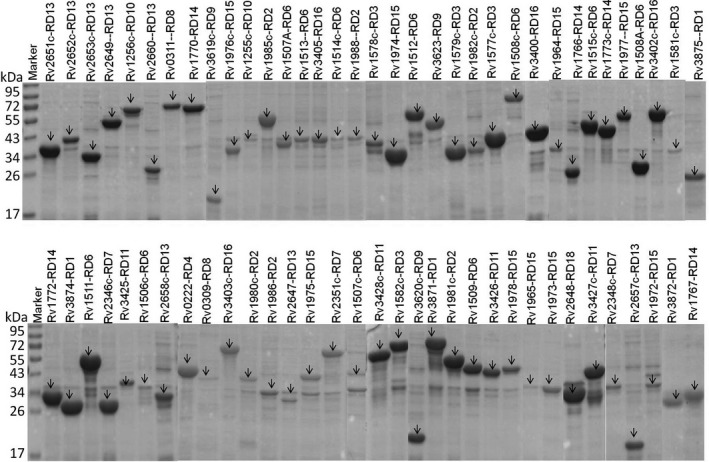
Examination of purified recombinant proteins from RD regions with SDS‐PAGE. All 68 purified protein samples were separated using 12% SDS‐PAGE in nine gels and Coomassie blue stained. The corresponding pictures are presented in combination. The protein size in bands is indicated by black arrows.

By using the 129 pairs of specific primers (Table S1), the 129 ORFs within 16 RDs were amplified with polymerase chain reaction (PCR). The accuracy of the insertion of RDs ORF into the recombinant pET‐32a was determined by digesting restrictively and sequencing commercially. The alignment between the cloned sequences and those in the *M. tuberculosis* genome (GenBank accession no: AL123456.3) was performed with BLAST on the National Centre for Biotechnology Information website. As a result, 75 ORFs were cloned with > 99% similarity at the nucleic acid level and 100% identity at the amino acid level. After induction, 68 ORFs were successfully expressed and purified (Fig. 1). These proteins were divided into eight categories according to function. The top five categories were hypothetical, phage, membrane/transmembrane, and secreted proteins, and transposase (Fig. S1A). Most of the recombinant proteins were expressed in inclusion bodies, while only seven proteins expressed in a soluble form, including Rv0311, Rv1766, Rv1767, Rv1976c, Rv2660c, Rv0222 and Rv3872.

### Initial screening of RD proteins with serum pools from TB patients

The 68 proteins were screened by the ratios of PTB‐to‐HC and EPTB‐to‐HC. As a result, 29 of the proteins were preliminarily selected for further evaluation with 19 proteins chosen by the ratio of PTB‐to‐HC and 13 proteins by the ratio of EPTB‐to‐HC (Table 1, Fig. S1B). Three proteins of the selected 29 proteins were shared by the PTB and EPTB groups (Rv0222, Rv3403c and Rv0309). In addition, five proteins (Rv3874, Rv3875, Rv3872, Rv1985c and Rv0222) were previously reported in other studies (Mukherjee *et al*., 2007; Rosenkrands *et al*., 2008; Chen *et al*., 2010).

**Table 1:** Selection of 29 recombinant proteins by serological initial screening and final confirmation

Proteins	Annotation	AUC^a^	95% CI	Ratio^b^
PTB versus HC				
Rv3403c	Hypothetical protein	0.8537	0.7779–0.9294	3.04
Rv0222	Probable enoyl‐CoA hydratase EchA1	0.8129	0.7280–0.8979	2.01
Rv3875	6 kDa early secretory antigenic target EsxA	0.7429	0.6565–0.8492	2.20
Rv0309	Possible conserved export protein	0.7374	0.6407–0.8340	2.56
Rv2657c	Probable PhiRv2 prophage protein	0.7353	0.6346–0.8360	2.50
Rv3872	PE family related protein PE35	0.7277	0.6302–0.8253	2.03
Rv2649	Probable transposase for insertion sequence element	0.7206	0.6202–0.8209	2.81
Rv1509	Hypothetical protein	0.7111	0.6129–0.8093	3.40
Rv3428c	Possible transposase	0.6894	0.5866–0.7923	2.24
Rv1256c	Probable cytochrome P450 130 Cyp130	0.6655	0.5641–0.7667	2.36
Rv2660c	Hypothetical protein	0.6590	0.5524–0.7657	2.09
Rv1573	Probable PhiRv1phage protein	0.6227	0.5337–0.7117	2.18
Rv3874	10 kDa culture filtrate antigen EsxB	0.6011	0.4916–0.7106	4.50
Rv3402c	Conserved hypothetical protein	0.5714	0.4623–0.6804	3.12
Rv3405c	Possible transcriptional regulatory protein	0.5690	0.4811–0.6569	2.40
Rv1972	Conserved Mce associated membrane protein	0.5562	0.4481–0.6642	2.53
Rv1981c	Ribonucleside‐diphpsphate reductase	0.5517	0.4422–0.6612	2.51
Rv3427c	Possible transposase	0.5447	0.4371–0.6523	2.58
Rv1964	Conserved hypothetical integral membrane protein	0.5381	0.4276–0.6486	2.70
EPTB versus HC				
Rv0222	Probable enoyl‐CoA hydratase EchA1	0.7523	0.5745–0.9302	2.04
Rv3403c	Hypothetical protein	0.7386	0.5974–0.8791	3.35
Rv1514c	Conserved hypothetical protein	0.6653	0.5474–0.7832	3.32
Rv2652c	Probable PhiRv2 prophage protein	0.6150	0.4885–0.7416	2.03
Rv2658c	Possible PhiRv2 protein	0.6141	0.4903–0.7379	2.74
Rv1577c	Probable PhiRv1phage protein	0.5946	0.4687–0.7205	2.60
Rv1767	Conserved protein	0.5722	0.4433–0.7010	2.47
Rv1508c	Probable membrane protein	0.5468	0.4195–0.6741	3.34
Rv0309	Possible conserved export protein	0.5464	0.4137–0.6791	2.04
Rv1255c	Probable transcriptional regulatory protein	0.5243	0.3915–0.6570	2.10
Rv1770	Conserved protein	0.5187	0.3869–0.6505	2.03
Rv3400	Probable hydrolase	0.5098	0.3791–0.6405	2.72
Rv1985c	Probable transcriptional regulatory protein	0.5044	0.3767–0.6321	3.55

**a**. AUC values with the 95% CI were used to confirm the potential diagnostic markers obtained by the initial screening with ELISA.

**b**. The ratio of OD_630_ between PTB (EPTB) and healthy control (HC) when the indirect ELISA was used to initially screen the potential diagnostic markers.

### Confirmation of application of 29 proteins in differentiation of TB from HC

**Figure 2:**
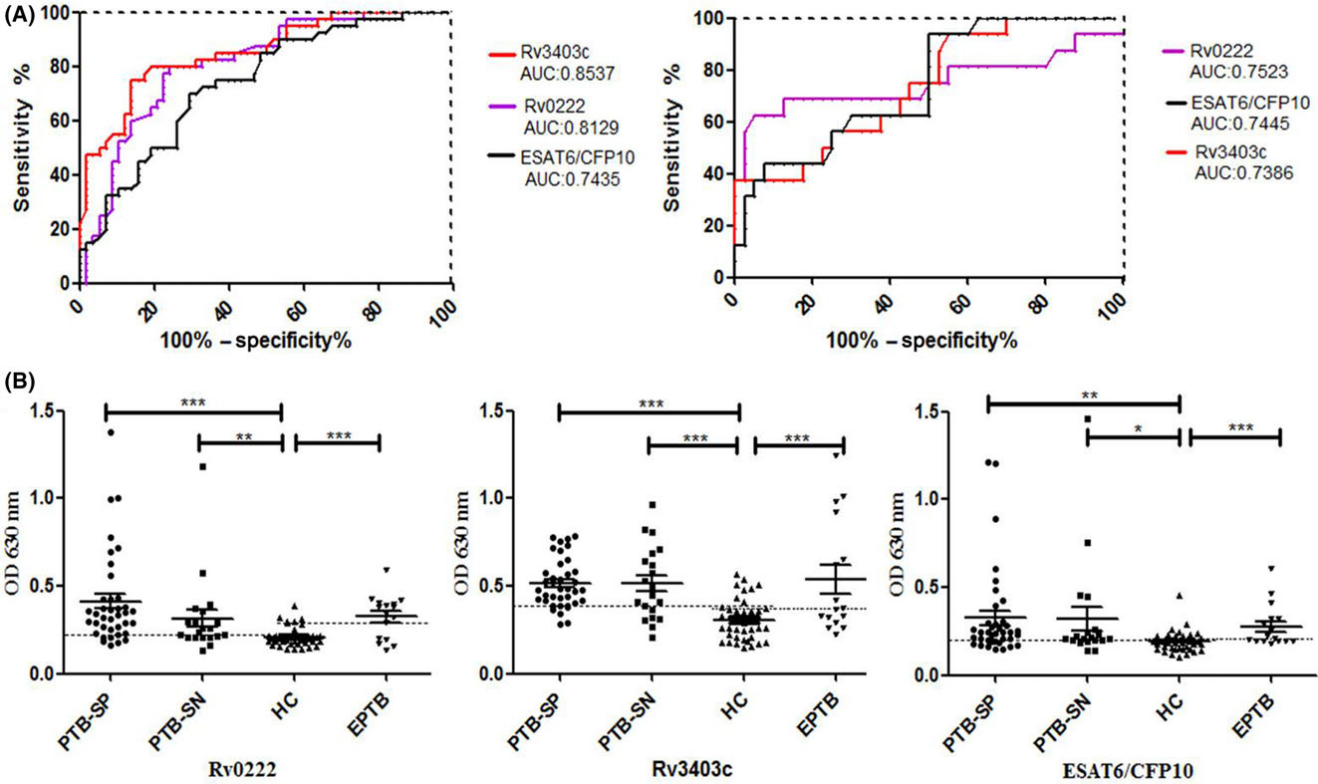
Levels of antibodies among different groups of PTB patients, EPTB patients and HC. A. ROC curve analysis of Rv3403c and Rv0222 performance using ESAT6/CFP10 as the positive control for serodiagnosis of PTB patients and HC control (left) or EPTB and HC control (right) was performed. B. Each point represents one serum sample. The horizontal solid lines on each group indicate the median value. Dotted lines on each group indicate the cut‐off value. The optimal cut‐off value representing maximum sensitivity and specificity (minimal false negative and positive rates) is determined by ROC curve analysis for serodiagnosis of PTB (EPTB) patients and HC. OD630 represents the optical density at a wavelength of 630 nm. The *P* values of the OD_630_ difference between each two groups were shown above the plots determined with one‐way ANOVA Newman‐Keels test. ***, *P *<* *0.001; **, *P *<* *0.01; *, *P *<* *0.05.

Twenty‐nine antigenic proteins were selected to be further evaluated with additional clinical samples. The ESAT6/CFP10 fusion protein was designated as the reference antigen. We performed receiver operating characteristic (ROC) analysis depending on antibody responses of sera from PTB (EPTB) and HC, then the results for the area under the curve (AUC) values of all 29 potential antigens were obtained (Table 1). Compared to ESAT6/CFP10 [AUC, 0.7435; 95% confidence interval (CI), 0.6465–0.8406], Rv0222 (RD4) and Rv3403c (RD16) were subjected to further confirmation with higher AUCs [0.8129 (95% CI, 0.7280–0.8979) and 0.8537 (95% CI, 0.7779–0.9294)] for the diagnosis of PTB. Rv0222 had the highest AUC value [0.7523 (95% CI, 0.5745–0.9302)] for the diagnosis of EPTB (Fig. 2A).

Then, we compared the sensitivities and specificities of the proteins between patient groups and the HC. The commercial TB‐DOT (Upper Biotech, Shanghai, China) test was used for comparison. The diagnostic sensitivities of TB‐DOT in the PTB and EPTB groups were 70.6% (41/58) and 62.5% (10/16), respectively, while the specificity was 85% (34/40).

The median IgG reactivity to Rv0222, Rv3403c, and ESAT6/CFP10 proteins was stronger in PTB‐SP, PTB‐SN, and EPTB than in HCs (Fig. 2B; *P *<* *0.05). The sensitivities of the proteins were slightly higher in PTB‐SP than PTB‐SN and EPTB (Table 2). With respect to PTB‐SP diagnosis, ESAT6/CFP10 had lower sensitivity (71.1%) and specificity (70%) than Rv0222 (86.8%/80%) and Rv3403c (86.8%/80%). In addition, the coincidence rates between Rv0222 and ESAT6/CFP10, between Rv0222 and TB‐DOT, between Rv3403c and ESAT6/CFP10, and between Rv3403c and TB‐DOT were 63.2% (24/38), 68.4% (26/38), 68.4% (26/38), and 73.7% (28/38), respectively. With respect to PTB‐SN diagnosis, Rv3403c exhibited a higher sensitivity (70%) than the other proteins. The coincidence rates between Rv3403c and ESAT6/CFP10 and between Rv3403c and TB‐DOT were 60% (12/20) and 40% (8/20), respectively. With respect to EPTB diagnosis, Rv0222 had a higher sensitivity (68.8%) than other antigens. The coincidence rates between Rv0222 and ESAT6/CFP10 and between Rv0222 and TB‐DOT were 62.5% (10/16) and 56.2% (9/16), respectively (Table S2). In addition, the highest sensitivity was obtained in abdominal TB [80% (4/5)] and lymph TB [75% (3/4)], while the lowest sensitivity was in pleural TB [40% (2/5)].

**Table 2:** Diagnostic sensitivities and specificities of antigen combinations

Groups /antigens	Sensitivity (%)	Specificity (%)	PPV (%)	NPV (%)
PTB‐SP versus HC				
A	86.8 (33/38)	80 (32/40)	80.5 (33/41)	86.5 (32/37)
B	86.8 (33/38)	80 (32/40)	80.5 (33/41)	86.5 (32/37)
C	71.1 (27/38)	70 (28/40)	69.2 (27/39)	71.8 (28/39)
A+B	94.7 (36/38)	72.5 (29/40)	76.6 (36/47)	93.5 (29/31)
A+C	94.7 (36/38)	70 (28/40)	75 (36/48)	93.3 (28/30)
B+C	92.1 (35/38)	62.5 (25/40)	70 (35/50)	89.3 (25/28)
A+B+C	94.7 (36/38)	62.5 (25/40)	70.6 (36/51)	92.6 (25/27)
TB‐DOT	76.3 (29/38)	85 (34/40)	82.9 (29/35)	79 (34/43)
PTB‐SN versus HC				
A	55 (11/20)	80 (32/40)	57.9 (11/19)	78 (32/41)
B	70 (14/20)	80 (32/40)	63.6 (14/22)	84.2 (32/38)
C	65 (13/20)	70 (28/40)	52 (13/25)	80 (28/35)
A+B	85 (17/20)	72.5 (29/40)	60.7 (17/28)	90.6 (29/32)
A+C	85 (17/20)	70 (28/40)	58.6 (17/29)	90.3 (28/31)
B+C	90 (18/20)	62.5 (25/40)	54.5 (18/33)	92.6 (25/27)
A+B+C	90 (18/20)	62.5 (25/40)	54.5 (18/33)	92.6 (25/27)
TB‐DOT	60 (12/20)	85 (34/40)	66.7 (12/18)	81 (34/42)
EPTB versus HC				
A	68.8 (11/16)	87.5 (35/40)	68.8 (11/16)	87.5 (35/40)
B	56.2 (9/16)	75 (30/40)	47.4 (9/19)	81.8 (30/37)
C	56.2 (9/16)	75 (30/40)	47.4 (9/19)	81.8 (30/37)
A+B	87.5 (14/16)	72.5 (29/40)	56 (14/25)	93.5 (29/31)
A+C	81.3 (13/16)	72.5 (29/40)	54.2 (13/24)	90.6 (29/32)
B+C	81.3 (13/16)	60 (24/40)	44.8 (13/29)	88.9 (24/27)
A+B+C	93.7 (15/16)	60 (24/40)	48.4 (15/31)	96 (24/25)
TB‐DOT	62.5 (10/16)	85 (34/40)	62.5 (10/16)	85 (34/40)

A, Rv0222; B, Rv3403c; C, ESAT6/CFP10; EPTB, Extra‐pulmonary TB; HC, Healthy control; PTB‐SP, Smear‐positive pulmonary TB; PTB‐SN, Smear‐negative pulmonary TB.

Sensitivity (%) = number of patients positive by ELISA/total number of confirmed patients × 100. specificity (%) = 100 – number of control samples positive by ELISA/total number of control samples × 100. PPV (positive predictive value) % = number of the true‐positive samples/ sum of true‐and‐false positive samples × 100. NPV (negative predictive value) % = number of true‐negative samples /sum of true‐and‐false negative samples × 100.

Then, we performed parallel tests by combining two and three protein‐based analyses. The sensitivity, specificity, positive predictive value (PPV), and negative predictive value (NPV) were achieved for PTB‐SP, PTB‐SN and EPTB groups (Table 2). The sensitivities were improved, while the specificities were similarly decreased. Taken together, only the Rv0222 and Rv3403c combination could improve the sensitivity of PTB‐SN‐to‐HC to 85% (17/20), while the specificity was maintained at 72.5% (29/40; Table 2).

### Test of potential application of Rv0222 and Rv3403c in differentiation of TB from LTBI

Antibody responses against Rv0222, Rv3403c and ESAT6/CFP10 proteins in the TB and latent *M. tuberculosis* infection (LTBI) groups were also compared. Although the median IgG responses to Rv0222, Rv3403c, and ESAT6/CFP10 proteins were higher in PTB‐SP, PTB‐SN, and EPTB than LTBI, there were no significant differences (*P *>* *0.05) except ESAT6/CFP10 (Fig. S2).

### Comparison of IgG antibody responses to Rv0222 and Rv3403c in the initial PTB and relapse PTB sub‐groups

**Figure 3:**
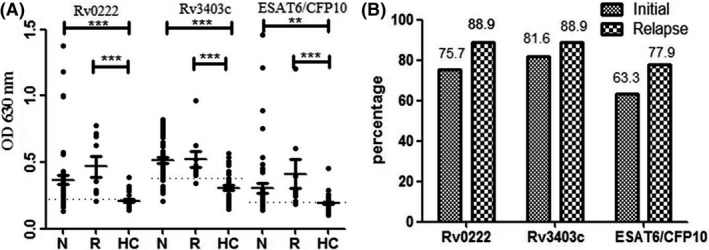
Levels of antibodies among different sub‐groups of initial and relapse patients. A. N, initial PTB; R, relapse PTB. The horizontal solid lines on each group indicate the median value. Dotted lines on each group indicate the cut‐off value. The *P* values of the OD_630_ difference between each of two groups were shown above the plots determined with one‐way ANOVA Newman–Keuls test. ***, *P *<* *0.001; **, *P *<* *0.01; (B) Percentages of initial PTB patients and relapse PTB patients with positive antibody responses to these three antigens.

We evaluated the IgG antibody responses against Rv0222, Rv3403c, and ESAT6/CFP10 proteins in the initial PTB and relapse PTB subgroups. The antibody levels against Rv0222, Rv3403c and ESAT6/CFP10 proteins were significantly higher in the initial PTB and relapse PTB patients than the HC (*P *<* *0.001 or *P *<* *0.01; Fig. 3A). In addition, the relapse PTB group had slightly higher positive rates than the initial PTB group for antibodies against all three proteins (Fig. 3B); however, there was no significant difference (*P *>* *0.05). Besides, there was no significant difference in antibody responses against these three proteins between the different age groups from TB (Fig. S3).

### Validation of Rv0222 and Rv3403c antigenicity in mice and humans

**Figure 4:**
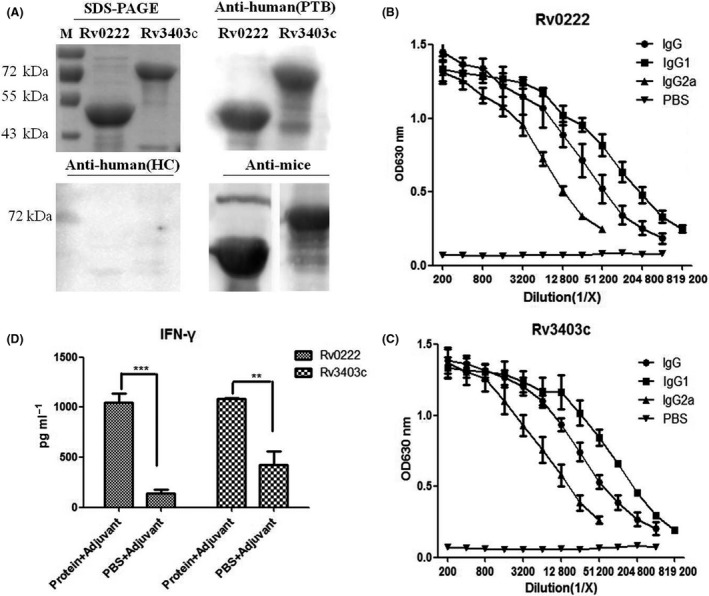
Levels of cellular and humoural immunity induced by Rv0222 and Rv3403c in mice. BALB/c mice were injected subcutaneously with 50 μg of each purified recombinant protein (or PBS) mixed with Freund's adjuvant. The mouse serum samples were collected after immunization.A. Western blotting analysis of two proteins with different sera. Purified recombinant proteins Rv0222 and Rv3403c were separated with a 12% SDS‐PAGE gel (upper left). M: reference proteins with the molecular mass labeled on the left; the other 3 pictures showed western blotting analysis of the two proteins with antisera from PTB patients, HC, and mouse antisera after immunization; (B) and (C): The mouse serum samples were analyzed with iELISA for the presence of anti‐Rv0222 (B) and anti‐Rv3403c(C) IgG, IgG1, and IgG2a. Each point represents the mean data from three individual mice with an error bar of SD; (D) Splenocytes (2 × 10^6^ cells/well) were stimulated with Rv0222 or 3403c (15 μg ml^−1^) for 48 h at 37°C. The cell supernatants were collected, IFN‐γ levels were measured with a commercial ELISA kit.

The antigenicity of the identified proteins (Rv0222 and Rv3403c) was evaluated with the sera from immunized mice and clinical samples from humans by western blotting assay (Fig. 4A). Strong specific reactions of recombinant proteins against the corresponding mouse antisera were observed after three vaccinations. The total IgG titers measured with iELISA were 100 × 2^11^ (Fig. 4B, C). The high IgG1 and IgG2a antibody titres were also detected in the sera of mice immunized with these two antigens, although the titres of IgG_1_were higher than IgG2a. These results indicated that both proteins were of good antigenicity and capable of stimulating the mice to produce high titers of specific antibodies. Furthermore, both proteins reacted specifically to the PTB serum pool, but not to the HC serum pool (Fig. 4A), demonstrating that Rv0222 and Rv3403c exhibited excellent immunoreactivities to human sera.

In addition, splenocytes from the protein‐immunized mice and PBS mock‐inoculated mice were stimulated with the recombinant antigens. IFN‐γ secretion by mouse spleen cells was detected with sandwich ELISA (Fig. 4D). IFN‐γ production (1.04 and 1.08 ng ml^−1^) in response to stimulation with Rv0222 (15 μg ml^−1^) and Rv3403c (15 μg ml^−1^) in the immunized mice, respectively, was significantly higher than that in negative control mice (0.13 and 0.42 ng ml^−1^, respectively; *P* <0.01). These results indicated that Rv0222 and Rv3403c evoked high cellular immunological responses.

## Discussion

In RDs, some antigens were reported to be potential markers for improving the TB serological diagnosis. ESAT‐6 (Rv3875), Rv3872, CFP‐10 (Rv3874) and Rv3879 in RD1 (Dillon *et al*., 2000; Mukherjee *et al*., 2007; Liu *et al*., 2016), Rv1980c in RD2 (Zhu *et al*., 2012), Rv0222 in RD4 (Rosenkrands *et al*., 2008), Rv0310c in RD8, Rv1255c in RD10 (Luo *et al*., 2017), Rv3425 in RD11 (Zhang *et al*., 2007), and Rv2645 in RD13 (Luo *et al*., 2015) have shown potential as TB diagnostic antigens. The genes in RD1, RD2 and RD8 have been reported to be conserved in both *M. tuberculosis* and *Mycobacterium bovis* (*M. bovis*), but are absent in *M. bovis* BCG, as well as several non‐tuberculosis mycobacteria (NTM), including *Mycobacterium avium* (Parkash *et al*., 2009). The genes in RD4, RD10, RD11 and RD13 were absent from most *M. bovis* and *M. bovis* BCG. Therefore, these antigens in RDs are potential serodiagnostic markers to distinguish active TB from BCG vaccination and non‐TB diseases in clinical practice. The sensitivities and specificities of various *M. tuberculosis* antigens ranged from 3% to 100% and from 51% to 100%, respectively (Parkash *et al*., 2009).

Compared to PTB‐SP diagnosis, PTB‐SN and EPTB diagnosis is difficult because the negative rate of sputum smear microscopy is very common and the pathological test samples are difficult to obtain. It has been reported that ~13% of initial TB infections are caused by PTB‐SN (Tostmann *et al*., 2008). Although the advent of Xpert MTB/RIF represents an important advance in PTB‐SN and EPTB diagnosis, the high price prevents Xpert MTB/RIF from widespread application and a true point‐of‐care test (Norbis *et al*., 2014). Thus, a rapid, affordable and accurate point‐of‐care method for TB diagnosis is needed. A new TB serodiagnosis with high sensitivity and specificity at a significantly reduced cost remains to be developed, and is especially true for the detection of PTB‐SN and EPTB.

In this study, the antibody levels against 68 recombinant antigens of *M. tuberculosis* in RD regions of serum samples from five different groups of PTB‐SP, PTB‐SN, EPTB, LTBI and HC were evaluated. The 29 proteins from initial screening were subjected to further confirmation. When the AUC values were compared, the top two proteins (Rv3403c and Rv0222) were selected and ESAT6/CFP10 served as a reference protein, with AUC values of 0.8537 (*P *<* *0.001), 0.8129 (*P *<* *0.001), and 0.7435 (*P *<* *0.001). Indeed, the Rv3403c and Rv0222 proteins had high sensitivities and specificities in discriminating PTB from HC. Besides, western blotting analysis showed that Rv0222 and Rv3403c reacted specifically to the PTB serum pool, but not to the HC serum pool. Therefore, it was concluded that Rv0222 and Rv3403c could be used as diagnostic markers to detect PTB patients. In addition, there was no significant difference in antibody response between young TB patients and older TB patients, which indicated that both marker proteins were suitable for different age groups; however, we found that the serum antibody levels against Rv0222 and Rv3403c could not differentiate PTB (EPTB) from LTBI. Hence, both markers should be used in combination with the clinical diagnosis, such as chest X‐ray images based on LTBI without symptoms in the clinical application of a future test.

As is known, ESAT6/CFP10 fusion protein has a high diagnostic value for TB screening. The sensitivities and specificities of serodiagnostic tests based on this antigen have been reported to range from 43% to 100% and from 44% to 100% (Parkash *et al*., 2009). We observed ~70% sensitivity and 70% specificity, with a good diagnostic value (AUC, 0.7435; *P *<* *0.001) for ESAT6/CFP10 antigen as a positive control, which was similar to the report of Wu *et al*. (Wu *et al*., 2010b).

Rv0222, a 263‐amino acid protein, is possibly an enoyl‐CoA hydratase (GenBank accession no: NP_214736), which is conserved in most mycobacterial species. Although Rv0222 was previously used to detect TB, with a sensitivity of 82% in 38 TB cases, including 29 PTB and 9 EPTB cases (Rosenkrands *et al*., 2008), no sensitivity to EPTB diagnosis was reported because of the small number of cases. Therefore, the present study achieved progress in applying Rv0222 for the detection of EPTB patients, especially the detection of abdominal TB and lymph TB. Our findings need to be confirmed with more samples.

Rv3403c, a 533 amino acid protein (GenBank accession no: NP_217920), is a hypothetical protein. Rv3403c is located in the RD16 region, and is absent from BCG‐Moreau strain and most NTM. Although in China the BCG Shanghai D2 strain (progeny strains of the Danish BCG‐823 strain) has been used in human vaccination, our results demonstrated that this protein could be used to differentiate TB patients from BCG‐vaccinated individuals. Whether or not Rv3403c might be expressed at different levels between BCG Shanghai D2 strain and *M. tuberculosis* remains to be investigated in the future. Furthermore, several previous reports have shown that diagnostic antigens, such as Ag85c (Samanich *et al*., 2000) and LAM (Wu *et al*., 2010a), are preferred to detect PTB‐SP with significantly higher detection rates than PTB‐SN. In the current study, we showed that Rv3403c had similarly high levels of detection (*P *>* *0.05) for PTB‐SP and PTB‐SN. Therefore, Rv3403c has promising application, especially in PTB‐SN detection.

The coincidence rates of Rv0222, Rv3403c and ESAT6/CFP10 proteins in the diagnosis of PTB‐SP, PTB‐SN, and EPTB groups were not high, indicating that immune recognition of antigens varies in different patients, and that no definite antigen was identified in all or a majority of samples (Lyashchenko *et al*., 1998). Thus, Liu *et al*. combined Rv3871, Rv3876, and Rv3879 proteins and improved the diagnostic ability in PTB‐SN(Liu *et al*., 2016). In our study, parallel tests by combining two and three antigens among Rv0222, Rv3403c and ESAT6/CFP10 were similarly investigated to improve the sensitivity of TB diagnosis. Unfortunately, although most combinations of two and three proteins displayed higher sensitivities, the specificities were substantially decreased. The combination of Rv0222 and Rv3403c had the highest value (85% + 72.5%) of sensitivity and specificity among other combinations of two or three proteins and any individual proteins in the group of PTB‐SN to HC. Therefore, the combination of Rv0222/Rv3403c in the fusion protein or antigens in the cocktail would be better serodiagnostic markers than individual proteins.

The B‐ and T‐ cell epitopes of both proteins were predicted using DNASTAR software and IEDB online software, as described previously (Wang *et al*., 2014; Paul *et al*., 2015). Rv0222 has two predicted B‐cell epitopes and three T‐cell epitopes, while Rv3403c has two B‐cell epitopes and five T‐cell epitopes (Table S3 and Fig. S4). There were no similar B‐ and T‐ cell epitopes between these two proteins. The IgG responses in mice and IFN‐γ production by mouse splenocytes to Rv0222 and Rv3403c proteins confirmed the epitope prediction, which indicates that these two proteins could evoke strong humoural and cell‐mediated (IFN‐γ) immune responses in mice, thus demonstrating more advantages of both proteins in future applications; however, the titres of IgG1 were higher than IgG2a, thus showing a Th 2‐biased immune response.

There were some limitations in this study. First, the samples tested could be increased in further studies to better validate the applications of these two proteins. Second, this study only used serum samples. Whether or not these findings could be extended to other types of samples, such as urine, saliva, and pleural fluid needs to be investigated in the future.

In conclusion, we have identified two diagnostic biomarkers (Rv0222 and Rv3403c) of *M. tuberculosis* which can be used for TB diagnosis, especially for the early diagnosis of PTB‐SN with Rv3403c and EPTB with Rv0222 or a combination of both for PTB‐SN.

## Materials and methods

### Ethics statement

The mouse experiments to produce antisera against the *M. tuberculosis* proteins were in strict accordance with the Hubei Regulations for Administration of Affairs Concerning Experimental Animals, and approved and supervised by the Scientific Ethical Committee for Experimental Animals of Huazhong Agricultural University (Permit Number: HZAURAB‐2015‐006).

The human blood samples were collected from healthy donors and TB patients or persons with LTBI by the physicians from Wuhan Medical Treatment Center during the services of physical examinations or patient treatment in accordance with the Measures for the Ethical Review of Biomedical Research Involving Human Subjects issued by the National Health and Family Planning Commission of The People's Republic of China and approved by the Ethics Committee of Wuhan Medical Treatment Center (#2015001). Written informed consent was provided by all participants involved in the study.

### Serum collection from human donors

A total of 156 HIV‐negative volunteers were recruited and classified into four groups, including 58 PTB patients and 16 EPTB patients, 42 LTBI persons, and 40 HCs. In the PTB group, 38 samples were from PTB‐SP patients, while 20 samples were from PTB‐SN patients. Among the PTB‐SP sub‐group, there were nine relapse PTB patients and 29 initial PTB patients. In the EPTB group, four samples were infected with lymph TB, five abdominal TB, five pleural TB and two meningitis TB. The characteristics of the study population are shown in Table 3.

**Table 3:** The demographic characteristics of the total study populations

Groups/Sub‐groups			Subjects (*n*)	Age (years)
PTB			58	39.12 ± 17.62
	PTB‐SP	Initial	29	35.70 ± 15.70
	PTB‐SP	Relapse	9	55.44 ± 17.74
	PTB‐SN	Initial	20	35.47 ± 16.13
EPTB	/	Initial	16	42.93 ± 16.63
LTBI	/	/	42	21.33 ± 2.37
HC	/	/	40	21.23 ± 2.08

EPTB, Extra‐pulmonary TB; HC, Healthy control; LTBI, Latent TB Infected; PTB‐SP, Smear‐positive pulmonary TB; PTB‐SN, Smear negative pulmonary TB; /, No reference.

Data are presented as the mean ± SD.

The blood samples were collected from the Wuhan Medical Treatment Center in China. The PTB cases were subjected to the conventional tests, including fever, cough and productive sputum, positive or negative acid‐fast bacilli on sputum smears, a suggestive chest X‐ray, and confirmed by *M. tuberculosis* isolation from sputum culture. EPTB was determined by clinical and radiographical findings, histopathological examination, and culture of biopsy tissue samples. All EPTB and 29 PTB patients underwent the initial diagnosis and treatment. Nine relapse PTB patients were considered cured after following a period of treatment or the initial treatment failed due to self‐discontinuation of medications and other reasons, but these nine patients had again developed smear‐positive bacteriologically active PTB. The samples from the LTBI and HC groups were obtained from student volunteers and the corresponding data were collected during physical examinations. The samples that were positive for IFN‐γ in the IGRA (QuantiFERON‐TB Gold; Neobioscience Technology, Shenzhen, China) with stimulation of ESAT6/CFP10 and no clinical symptoms belonged to the LTBI group. The HC was negative for all tests, including TST (induration area < 5 mm), clinical tests, and blood IFN‐γ tests.

### Cloning, expression, and purification of RD antigens

All 129 genes in the RDs (RD1‐16) were amplified with PCR and expressed in *Escherichia coli* as His fusion proteins, the details of which are included in Supporting information.

### Screening of seroreactive proteins with iELISA

The iELISA involved coating the recombinant proteins, and was established for screening, as described subsequently. The iELISA based on the recombinant fusion protein, ESAT6/CFP10, was prepared by this laboratory and designated as the positive control for TB antibody testing. Because the recombinant proteins were expressed in *E. coli*, each 100 μl of serum was first absorbed for 2 h at room temperature by adding 2 μl lysate of *E. coli* transformed with blank vector pET‐32a (Novagen, Madison, WI, USA) to remove potential false‐positive responses to *E. coli* components from serum samples.

In the initial screening, we used three different sera from PTB, EPTB and HC groups to react with the purified proteins. Each sera pool had five samples from the same group. The purified proteins were diluted to 500 ng ml^−1^ with coating buffer [0.05 M Na_2_CO_3_‐NaHCO_3_ (pH 9.6)], and 0.1 ml from each diluted protein was added to each well in a 96‐well plate (Thermo Fisher Scientific, Shanghai, China) and incubated overnight at 4°C. After each incubation, the plates were washed three times with 0.3 ml of PBST (137 mM NaCl, 2.7 mM KCl, 10 mM Na_2_HPO_4_, 2 mM KH_2_PO_4_, and 0.05% Tween‐20), then blocked for 1 h at 37°C with 0.2 ml 5% skim milk dissolved in PBST. A 0.1 ml aliquot of each absorbed serum sample (1:100 dilution in PBST) was added to each well in triplicate and incubated for 30 min at 37°C. After incubation and rewashing, 100 μl of horseradish peroxidase (HRP) conjugated goat‐anti‐human IgG (H  +  L) (1:10 000 dilution; Pierce, Waltham, MA, USA) was overlaid for 30 min at 37°C. Then, the reaction proceeded by adding 0.1 ml of tetramethylbenzidine /H_2_O_2_ substrate solution for 10 min at room temperature in the dark and was stopped by adding 50 μl of hydrofluoric acid. The optical density at 630 nm (OD_630_) was then measured. The OD_630_ ratio of PTB‐ or EPTB‐to‐HC group ≥ 2‐fold was designated as positive, and the proteins were subjected to further confirmation.

For further confirmation, each protein was coated in 96‐well plates and to reaction to individual serum samples from all three groups (PTB, EPTB and HC) in triplicate was detected with iELISA. ROC curves were constructed by plotting the true‐positive rate (sensitivity) and false‐positive rate (1‐specificity) with antibody responses of serum from PTB or EPTB patients and HCs against the recombinant proteins. The AUC values of proteins were compared with ESAT6/CFP10 and proteins with higher AUC values than ESAT6/CFP10 were selected. For the parallel test analysis, a sample was shown to be positive when an assay was considered to be positive, while a sample was negative when all assays were considered to be negative.

During the integrated analysis, antibody responses against the three proteins were also evaluated to verify the differences between PTB/EPTB and LTBI, and between initial and relapse PTB.

### Murine model: assessment of cellular and humoral immunity induced by proteins

Nine specific pathogen‐free female BALB/c mice (5–6 weeks old) were injected subcutaneously with 50 μg of each purified recombinant protein mixed with complete Freund's adjuvant (Sigma, Shanghai, China) as the initial immunization. Nine mice were immunized by two proteins and PBS as a negative control with every three mice immunized by one protein or PBS. Mice were boosted twice with an equal amount of proteins mixed with incomplete Freund's adjuvant (Sigma) 2 weeks later. The antibody titers of sera collected prior to each immunization were detected. At the end of the experiment, the mice were killed to obtain splenocytes. The lymphocytes were plated at 2 × 10^6^ cells/well in 24‐well plates (Costar, Corning, NY, USA) in RPMI‐1640 medium containing 10% fetal calf serum. Endotoxin detection (Chinese Horseshoe Crab Reagent Manufactory, Xiamen, China) and removal of these proteins were performed as described previously (Luo *et al*., 2017). The cells were stimulated with purified recombinant proteins (15 μg ml^−1^) at 37°C for 48 h. After stimulation, the supernatants were collected, and IFN‐γ was detected using a mouse IFN‐γ ELISA Kit (Neobioscience).

### Western blot analysis

Each recombinant protein (40 μg) was separated using 12% sodium dodecyl sulfate–polyacrylamide gel electrophoresis (SDS‐PAGE), then transferred onto polyvinylidene fluoride (PVDF) membranes. PVDF membranes were blocked at 37°C in an incubator overnight with 5% (w/v) skim milk dissolved in TBST [20 mM Tris‐base (pH 7.5), 150 mM NaCl, and 0.05% Tween‐20], then washed three times with TBST. After washing, PVDF membranes were incubated with mouse antisera (1:400 dilution) against the recombinant proteins or mixed sera diluted in TBST (1:200 dilution) from PTB patients, or mixed sera from the HC (1:200 dilution). After incubation and rewashing, PVDF membranes were re‐incubated at 37°C for 1 h with HRP‐conjugated goat anti‐mouse IgG/IgG1/IgG2a (1:5000 dilution; SouthernBiotech, Birmingham, AL, USA) and HRP‐conjugated goat anti‐human IgG (1: 5000 dilution; Invitrogen, Carlsbad, CA, USA). The images of PVDF membranes were generated using a chemiluminescent substrate and observed on the Chemiluminescence & Fluorescence DNr Bio‐imaging system (DNR, Jerusalem, Israel).

### Statistical analysis

Sensitivity refers to the proportion of patients that tested positive, while specificity refers to the proportion of healthy individuals that tested negative. The antibody IgG levels against each protein in the PTB‐SP, PTB‐SN, EPTB, LTBI, and HC groups were plotted using GraphPad Prism V5 (GraphPad Software, San Diego, CA, USA). Statistical significance was calculated using a non‐parametric Mann–Whitney *U* test, and a *P *<* *0.05 was considered to be statistically significant. Test performance in terms of sensitivity and specificity was evaluated for each protein by a ROC curve. The accuracy of the test was determined by the AUC and the test is moderately accurate with 0.7 < AUC ≤ 0.9 (La Manna *et al*., 2018). The optimal cut‐off value of each protein was also determined with the ROC curve to maximal specificity and sensitivity. The PPV = the number of true‐positive samples/sum of true‐and false‐ positive samples, and the NPV = the number of true‐negative samples/sum of true‐and false‐ negative samples.

## Conflict of interest

None declared.

## Supporting information


**Fig. S1.** Classification of the antigenic proteins identified according to their annotations.

**Fig. S2.** Levels of antibody responses among different groups of EPTB patients, PTB patients and LTBI**.**

**Fig. S3.** Levels of antibody responses among different age groups of PTB patients and EPTB patients.

**Fig. S4.** B cell epitopes prediction using DNAstar software.

**Table S1.** Primers and enzymes used for cloning of RD proteins.
**Table S2.** The coincidence rates between identified antigens and ESAT6/CFP10, TB‐DOT in TB diagnosis.
**Table S3.** B‐and T‐cell epitopes of Rv0222 and Rv3403c.

**Data S1**. Materials and methods.
