# Supplementary figures and images for: Identification of new diagnostic biomarkers for Mycobacterium tuberculosis and the potential application in the serodiagnosis of human tuberculosis
Source: Microb Biotechnol. 2018 Jun 27;11(5):893–904. doi: 10.1111/1751-7915.13291 (PMC6116745; doi:10.1111/1751-7915.13291)

(A)

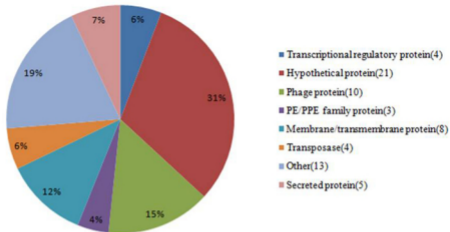

(B)

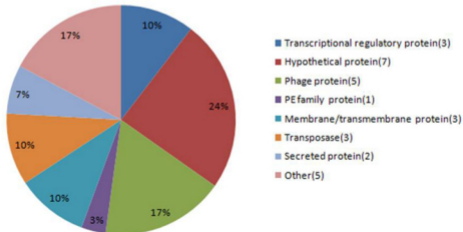

Supplement: Supplementary file 1 — Fig. S1. Classification of the antigenic proteins identified according to their annotations. [file MBT2-11-893-s001.pdf]

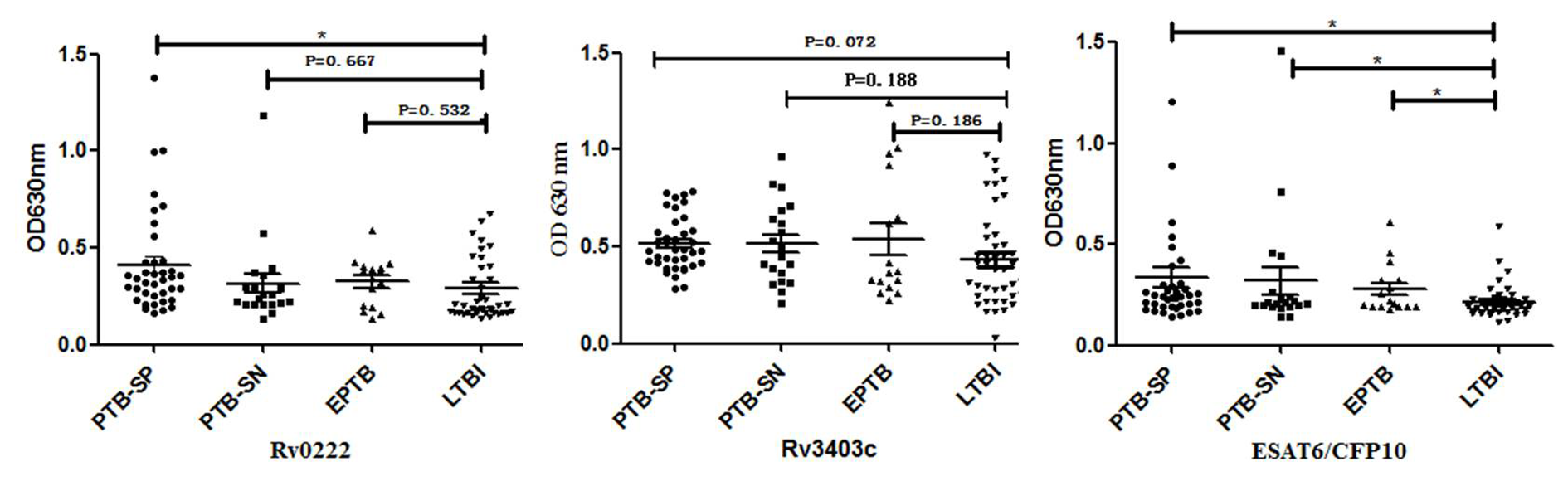

Supplement: Supplementary file 2 — Fig. S2. Levels of antibody responses among different groups of EPTB patients, PTB patients and LTBI. [file MBT2-11-893-s002.tif]

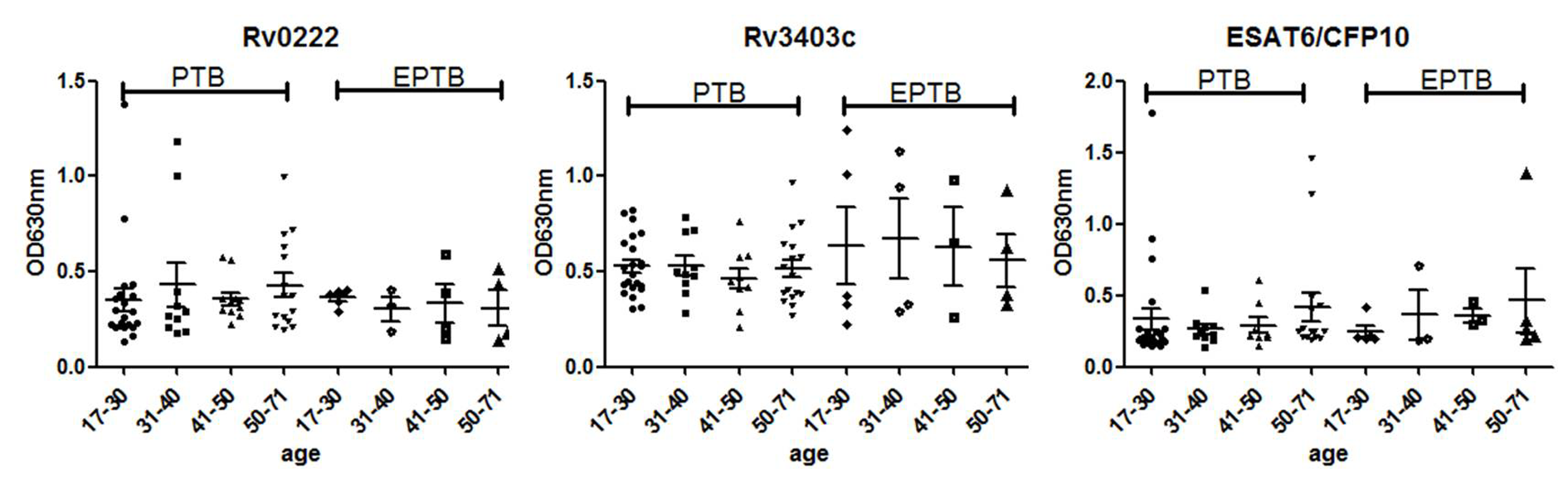

Supplement: Supplementary file 3 — Fig. S3. Levels of antibody responses among different age groups of PTB patients and EPTB patients. [file MBT2-11-893-s003.tif]

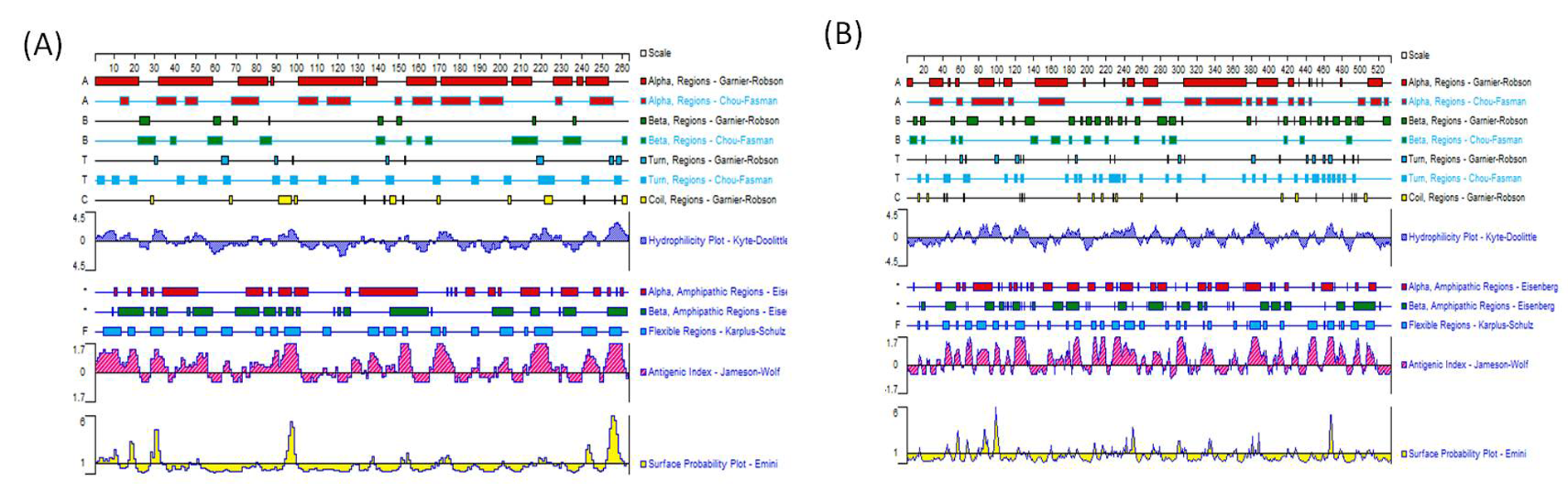

Supplement: Supplementary file 4 — Fig. S4. B cell epitopes prediction using DNAstar software. [file MBT2-11-893-s004.tif]
