# Supplementary material for: Identification of new diagnostic biomarkers for Mycobacterium tuberculosis and the potential application in the serodiagnosis of human tuberculosis
Source: Microb Biotechnol. 2018 Jun 27;11(5):893–904. doi: 10.1111/1751-7915.13291 (PMC6116745; doi:10.1111/1751-7915.13291)
Supplement: Supplementary file 5 — Table S1. Primers and enzymes used for cloning of RD proteins. Table S2. The coincidence rates between identified antigens and ESAT6/CFP10, TB‐DOT in TB diagnosis. Table S3. B‐and T‐cell epitopes of Rv0222 and Rv3403c. [file MBT2-11-893-s005.doc]

**Table S1** Primers and enzymes used for cloning of RD proteins

| RD region | Name | Primer |
| --- | --- | --- |
| RD1 | Rv3871F | GAAC*GGATCC*ATGACTGCTGAACCGGAAGTAC |
|  | Rv3871R | GATC*AAGCTT*TTAACCGGCGCTTGGGGGTGCT |
|  | Rv3872F | GCGC*GGATCC*ATGGAAAAAATGTCACATG |
|  | Rv3872R | GACT*AAGCTT*CTATTCGGCGAAGACGCC |
|  | Rv3873F | GTAT*GGATCC*ATGCTGTGGCACGCAATGCC |
|  | Rv3873R | GTCC*AAGCTT*TCACCAGTCGTCCTCTTCGT |
|  | Rv3874F | GAAT*GGATCC*ATGGCAGAGATGAAGACC |
|  | Rv3874R | GATC*AAGCTT*TCAGAAGCCCATTTGCGA |
|  | Rv3875F | TAAT*GGATCC*ATGACAGAGCAGCAGTGG |
|  | Rv3875R | GCAC*AAGCTT*CTATGCGAACATCCCAGT |
|  | Rv3876F | GAAC*GAATTC*ATGGCGGCCGACTACGACAAGCTC |
|  | Rv3876R | GAAC*AAGCTT*TCAACGACGTCCAGCCCTCTCGAA |
|  | Rv3877F | GAT*GGATCC*TTGAGCGCACCTGCTGTTGCTG |
|  | Rv3877R | CGT*AAGCTT*TCAGAACCGGATATTGCGGACC |
|  | Rv3878F | ATAT*GGATCC*ATGGCTGAACCGTTGGCCG |
|  | Rv3878R | GCGC*AAGCTT*CTACAACGTTGTGGTTGTTG |
|  | Rv3879F | GAAC*GAATTC*ATGAGTATTACCAGGCCGACGGGC |
|  | Rv3879R | TATA*AAGCTT*TCAGCATGCGGCGGCCAGGGCCCG |
| RD2 | Rv1978F | ATA*GGATCC*ATGGGTGAGGCGAACATCCG |
|  | Rv1978R | ATA*AAGCTT*TCATTCGCCGGGTTGGCGATC |
|  | Rv1979F | ATA*GGATCC*GTGGTCGGCCCGCGGACGAGAG |
|  | Rv1979R | AGC*AAGCTT*CTAGCGAGTGCTCGGCCGATCG |
|  | Rv1980F | ATA*GGATCC*GTGCGCATCAAGATCTTCATG |
|  | Rv1980R | AGC*AAGCTT*CTAGGCCAGCATCGAGTCG |
|  | Rv1981F | AGT*GGATCC*ATGACCGGCAAGCTCGTTGAG |
|  | Rv1981R | AGC*AAGCTT*TTAGAAGTCCCAGTCGGTGTC |
|  | Rv1982F | AGT*GGATCC*ATGATCGTGGACACAAGC |
|  | Rv1982R | AGC*AAGCTT*TTACGCGACGCCTGGCCAG |
|  | Rv1983F | AGT*GAATTC*GTGTCATTTCTGGTCGTGGTTCC |
|  | Rv1983R | AGT*AAGCTT*TCACGCCGGATGATCAAAGACTG |
|  | Rv1984F | AGT*GAATTC*ATGACTCCACGCAGCCTTG |
|  | Rv1984R | AGT*AAGCTT*TCATCCGGCGTGATCGAGC |
|  | Rv1985F | AGT*GAATTC*ATGGTGGATCCGCAGCTTG |
|  | Rv1985R | AGT*AAGCTT*TCAACCCGGTCGGCGGC |
|  | Rv1986F | AGT*GGATCC*GTGAACTCACCACTGGTCGT |
|  | Rv1986R | AGC*AAGCTT*CTAGGTCACGGTCAGCGAG |
|  | Rv1987F | AGT*GGATCC*ATGGCCGGACTGAACATTTAC |
|  | Rv1987R | AGT*Gagctc*CTAGGTGCAAGGATATTGC |
|  | Rv1988F | AGT*GGATCC*GTGTCCGCCCTCGGACGGTCG |
|  | Rv1988R | AGC*AAGCTT*TTACCGCCCCTGCCAGTCAC |
| RD3 | Rv1573F | AGT*GGATCC*ATGACCACCACACCAGCAC |
|  | Rv1573R | AGT*AAGCTT*TCAGCGCTCGTCTTCCGC |
|  | Rv1574F | AGT*GGATCC*ATGGGCTACAAACCAGAATC |
|  | Rv1574R | AGT*AAGCTT*CTACCTCCTTGTCAGTGTGG |
|  | Rv1575F | AGT*GGATCC*ATGGAACCAAAGCCGTCTC |
|  | Rv1575R | AGT*AAGCTT*TCAGATGTGCTGGTGCGC |
|  | Rv1576F | AGT*GGATCC*ATGACCGAATTCGACGACATC |
|  | Rv1576R | AGT*AAGCTT*CTACGCGGTAGTCTCCACCTTC |
|  | Rv1577F | AGT*GGATCC*ATGGCCGAGCTGCGGTCT |
|  | Rv1577R | AGT*AAGCTT*TCACCAATCGAGTAGGGACAG |
|  | Rv1578F | AGT*GGATCC*ATGCCAAGACCACCGAAAC |
|  | Rv1578R | AGT*AAGCTT*TCACCGGTCCGGGGCAAAC |
|  | Rv1579F | AGT*GGATCC*GTGACCCCGATCAACCGG |
|  | Rv1579R | AGT*AAGCTT*TCACGATGGCGACCCCG |
|  | Rv1580F | AGT*GGATCC*ATGGCTGAAACCCCCGAC |
|  | Rv1580R | AGT*AAGCTT*TCACTGGTCGACCTCTAT |
|  | Rv1581F | AGT*GGATCC*ATGACCGCTGTCGCGATCAC |
|  | Rv1581R | AGT*AAGCTT*TCAAGCAGGGTTGGTAAGTG |
|  | Rv1583F | AGT*GGATCC*ATGGCCGACATCCCCTACGGC |
|  | Rv1583R | AGT*AAGCTT*CTAATCATCGAAATCATCGGCC |
|  | Rv1584F | AGT*GGATCC*GTGTCGACCATCTACCATC |
|  | Rv1584R | AGT*AAGCTT*TCATCGGGCACCGCCTGAC |
|  | Rv1585F | AGT*GGATCC*ATGAGCCGGCACCACAACATC |
|  | Rv1585R | AGT*AAGCTT*CTAGCAGCTACCACGCGTTG |
|  | Rv1586F | AGT*GGATCC*GTGAGATACACTACACCTGTGCGTG |
|  | Rv1586R | AGT*AAGCTT*TCATCGCCAATTCACCTGCACCCGT |
| RD4 | Rv0221F | ata*ggatcc*GTGAAACGGCTCAGCGGCTGGGAC |
|  | Rv0221R | ata*cggccg*TCATGCCTGCGCCATCGCGGACTC |
|  | Rv0222F | ata*ggatcc*ATGAGCAGCGAAAGCGACG |
|  | Rv0222R | ata*aagctt*CTAGGTGCCCGTCCAACGG |
|  | Rv0223F | ata*ggatcc*ATGTCTGACAGTGCCACGGAATAC |
|  | Rv0223R | ata*cggccg*CTACGCGACGGTGTAGCCCATC |
| RD5 | Rv3117F | ata*gaattc*ATGGCACGCTGCGATGTCC |
|  | Rv3117R | atactcgagTCAGCTTCCCAACTCGATCGG |
|  | Rv3118F | ATA*GAATTC*ATGTGCTCTGGACCCAAGC |
|  | Rv3118R | ATA*AAGCTT*TCAGGTGATCTTGACGTCT |
|  | Rv3119F | ATA*GGATCC*ATGGCCAATGTGGTAGCTG |
|  | Rv3119R | ATA*AAGCTT*TCATGGTCTATCGCCGACC |
|  | Rv3120F | ATA*GAATTC*ATGAGTCCGTCTCCATCGGC |
|  | Rv3120R | ATA*AAGCTT*TCACAGTGACCGTTGGGCG |
|  | Rv3121F | ATA*GGATCC*ATGACAAGCACCTCGATTCCG |
|  | Rv3121R | ATA*AAGCTT*TCACGTCGGCCAGGTAACAAGG |
| RD6 | Rv1506F | ATA*GGATCC*GTGCGCATTGTCAATGCGG |
|  | Rv1506R | ATA*AAGCTT*TCATGACGCCTTCCTAACC |
|  | Rv1507AF | ATA*GGATCC*ATGCAATCAGGTCAAAAT |
|  | Rv1507AR | ATA*AAGCTT*TCAACCCGCTAGAAGGCCG |
|  | Rv1507CF | ATA*GGATCC*TTGAAGAAAGTCGCGATTGT |
|  | Rv1507CR | ATA*AAGCTT*TCACTGGCGACAGTACCTC |
|  | Rv1508AF | ATA*GGATCC*GTGAAGCGAGCGCTCATC |
|  | Rv1508AR | ATA*AAGCTT*TTACTCAACGCAAACACC |
|  | Rv1508CF | ATA*GGATCC*GTGATTCCGGTGATGAGCGCTCGCT |
|  | Rv1508CR | ATA*AAGCTT*CTATGGGGTGTAATTTTGGCAGATT |
|  | Rv1509F | ATA*GGATCC*GTGTTTGCGTTGAGTAATAATC |
|  | Rv1509R | ATA*AAGCTT*TTACCTCTTCGTTAGCCGCAC |
|  | Rv1510F | ata*GGATCC*ATGTACGAGAGACGGCATGAGCG |
|  | Rv1510R | ATA*AAGCTT*TCAGTCGGGTCGCCGCGCCAAC |
|  | Rv1511F | ATA*GAATTC*GTGAAGCGAGCGCTCATCACCG |
|  | Rv1511R | ATA*AAGCTT*TCATGTCCGGCCGGCGATC |
|  | Rv1512F | ATA*GAATTC*ATGAACGCGCACACCTCG |
|  | Rv1512R | ATA*AAGCTT*TCATTGCCGAACCGTTCC |
|  | Rv1513F | ATA*GGATCC*TGAGGCTGGCCCGTCGC |
|  | Rv1513R | ATA*AAGCTT*TCAATCGCTGCCCCGGAAG |
|  | Rv1514F | ata*GGATCC*GTGACGTCTGCTCCGACCG |
|  | Rv1514R | ATA*AAGCTT*CTATTTCGACATTCGCGTG |
|  | Rv1515F | ATA*GGATCC*ATGTCGACAAACCCAGGACC |
|  | Rv1515R | ATA*AAGCTT*CACCGGGTCTTGATACCGAT |
|  | Rv1516F | ATA*GAATT*CGTGAGTCCCCAGCTTTGCC |
|  | Rv1516R | ATA*AAGCTT*TCAGGCTCGACAGCCGC |
| RD7 | Rv2346F | ATA*GGATCC*ATGACCATCAACTATCAGTT |
|  | Rv2346R | ATA*AAGCTT*TCAGGCCCAGCTGGAGCC |
|  | Rv2347F | ATA *GAATTC* ATGGCAACACGTTTTATGAC |
|  | Rv2347R | ATA *AAGCTT* TTAGCTGCTGAGGATCTGC |
|  | Rv2348F | ATA*GGATCC*GTGCTTTTGCCTCTTGGT |
|  | Rv2348R | ATA*AAGCTT*CTAGCCGGCCGCCGGAG |
|  | Rv2349F | ATA*GGATCC*ATGTCACGCCGAGCATTCCTGG |
|  | Rv2349R | ATA*AAGCTT*CTAGCAGATGCCGCTGGGAATC |
|  | Rv2350F | ATA*GAGCTC*ATGACCCGCCGACAATTTTTTG |
|  | Rv2350R | ATA*AAGCTT*TCAACAGAGACCGCTGGGAATC |
|  | Rv2351F | ATA*GGATCC*ATGTCACGTCGAGAGTTTTTG |
|  | Rv2351R | ATA*AAGCTT*TCAGCTGCACAGCCCGCTG |
|  | Rv2352F | ATA*GGATCC*ATGATTTTGGATTTTTCGTG |
|  | Rv2352R | ATA*AAGCTT*CTATCCGATCCCGACCCG |
|  | Rv2353F | ATA*GGATCC*ATGCCGGGGCGGTTCAGAAAC |
|  | Rv2353R | ATA*AAGCTT*CTATCCGAACAAGTTCTTGAAG |
| RD8 | Rv0309F | ATA*GGATCC*ATGAGCCGACTCCTAGCTTTG |
|  | Rv0309R | ATA*AAGCTT*TTACTTGGCGATCGCGATC |
|  | Rv0310F | ATA*GGATCC*GTGTGCTGCAATGGCGTG |
|  | Rv0310R | ATA*AAGCTT*TCAATCGGCCAGCGCG |
|  | Rv0311F | ATA*GGATCC*ATGAGCCAATCCCGGTACGCG |
|  | Rv0311R | ATA*AAGCTT*TCATTTCACGGTCAACGGCAG |
|  | Rv0312F | ATA*GAATTC*ATGTACGACCCGCTGGGGTTGTC |
|  | Rv0312R | ATA*AAGCTT*TCACCCAGAGCCAAGGCTTCCG |
| RD9 | Rv3617F | ATA *GGATCC* ATGGGCGCACCTACCGAACGG |
|  | Rv3617R | ATA *AAGCTT* TCATCGCAACTCCAACCCCG |
|  | Rv3618F | ATA*GGATCC*ATGAAGGCACCGTTGCGTTTT |
|  | Rv3618R | ATA*AAGCTT*TCAGCTGCCCGCTTCCCCTT |
|  | Rv3619F | ATA*GGATCC*ATGACCATCAACTATCAATTC |
|  | Rv3619R | ATA*AAGCTT*TTAGGCCCAGCTGGAGCC |
|  | Rv3620F | ATA*GAATTC*ATGACCTCGCGTTTTATG |
|  | Rv3620R | ATA*AAGCTT*TCAGCTGCTGAGGATCTG |
|  | Rv3621F | ATA*GGATCC*ATGCTGGACTTTGCTCAGTT |
|  | Rv3621R | ATA*AAGCTT*TCATCCGCCAGCCGGCGGTTG |
|  | Rv3622F | ATA*GGATCC*ATGTCGATCATGCACGCC |
|  | Rv3622R | ATA*AAGCTT*CTAAGCGATCGTGGCGGC |
|  | Rv3623F | ATA*GAATTC*ATGATCCGATTGGTCCGTC |
|  | Rv3623R | ATA*AAGCTT*TAGGTCAGTTCCCAGACC |
| RD10 | Rv1255F | ATA*GGATCC*ATGGCGGGTACCGACTG |
|  | Rv1255R | ATA*AAGCTT*TCACTCGGGTCCAGGGT |
|  | Rv1256F | ATA*GAGCTC*ATGACATCAGTAATGTCTCACG |
|  | Rv1256R | ATA*AAGCTT*TCAGGATGTCACTCGGAACG |
|  | Rv1257F | ATA*GAATTC*GTGAATACCGATGTGCTGGCTG |
|  | Rv1257R | ATA*AAGCTT*CAGATCGCCGAGCCGGGATT |
| RD11 | Rv3425F | ATA*GGATCC*ATGCATCCAATGATACCAGC |
|  | Rv3425R | ATA*AAGCTT*CTACCCGCCCCTGTAGATC |
|  | Rv3426F | ATA*GGATCC*ATGCATCTAATGATACCCGC |
|  | Rv3426R | ATA*AAGCTT*CATCTCGGTTTCTCCTGAC |
|  | Rv3427F | ATA*GGATCC*ATGTCTATCTGTGATCCGG |
|  | Rv3427R | ATA*AAGCTT*CTAGCTGGTGGTGCGGC |
|  | Rv3428F | ATA*GGATCC*GTGGCCACGATAGCCCAACGGCT |
|  | Rv3428R | ATA*AAGCTT*TCAGGGCCGCGCAGGAACGCC |
|  | Rv3429F | ATA*GGATCC*ATGCATCCAATGATACCAGC |
|  | Rv3429R | ATA*AAGCTT*CTACCCGCCCCCGCCCCCGT |
| RD12 | Rv2072F | ATA*GGATCC*ATGATCATCGTTGTCGGGATCG |
|  | Rv2072R | ATA*AAGCTT*TCATCGCTTGGTCACCGACC |
|  | Rv2073F | ATA*GGATCC*GTGGACGACACGGGCGCT |
|  | Rv2073R | ATA*AAGCTT*TCATCGCGGCATCCTGCG |
|  | Rv2074F | ATA*GAATTC*GTGGCGATGGTCAACACC |
|  | Rv2074R | ATA*AAGCTT*TCAGGCCCGGTCGAGCAG |
|  | Rv2075F | ATA*GGATTC*TGCCCCGCGCCCGATGGC |
|  | Rv2075R | ATA*AAGCTT*TCATGGCGGCAGTAGGTAATGC |
| RD13 | Rv2645F | ATA*GGATCC*ATGACCACCACGCCCCGA |
|  | Rv2645R | ATA*AAGCTT*CACCGCCGGTGTTCGCC |
|  | Rv2646F | ATA*GGATCC*GTGAACACCGCGACCCGGGTC |
|  | Rv2646R | ATA*AAGCTT*TCACCATGCGGCTGCTGCTGC |
|  | Rv2647F | ATA*GGATCC*GTGCACGTGTGCCACAC |
|  | Rv2647R | ATA*AAGCTT*TCAGCCGCTCCAACCG |
|  | Rv2648F | ATA*GGATCC*ATGTCAGGTGGTTCATCG |
|  | Rv2648R | ATA*AAGCTT*TTAGCGTGCTGGCCGGTC |
|  | Rv2649F | ATA*GAATTC*AAAGACCGCGTCGGCTTTCTTC |
|  | Rv2649R | ATA*AAGCTT*TCAGCCGGCGGCTGGTCTCT |
|  | Rv2650F | ATA*GAATTC*ATGACTAATGAACAACATTTCG |
|  | Rv2650R | ATA*AAGCC*TCTACGCGGTGGTCTGCAC |
|  | Rv2651F | ATA*GGATCC*TTGAGTAGCATCCTTTTCC |
|  | Rv2651R | ATA*AAGTCC*TTACCAATCCATCAGGGCT |
|  | Rv2652F | ATA*GGATCC*TTGCCATCGCCAGCAACCG |
|  | Rv2652R | ATA*AAGTCC*TCACCGGTCTGGGGCGAAC |
|  | Rv2653F | ATA*GGATCC*TTGACCCACAAGCGCAC |
|  | Rv2653R | ATA*AAGTCC*TCACTGTTTGCTGTCGG |
|  | Rv2654F | ATA*GGATCC*ATGAGCGGCCACGCGTT |
|  | Rv2654R | ATA*AAGTCC*TCACGGCGGATCACCCCG |
|  | Rv2655F | ATA*GGATCC*ATGGCTGACATCCCCTACGG |
|  | Rv2655R | ATA*AAGTCC*TCATCGGGCGCCGTCCCGAG |
|  | Rv2656F | ATA*GGATCC*ATGACCGCCGTCGGCG |
|  | Rv2656R | ATA*AAGTCC*TCAGACCACCACCCGCC |
|  | Rv2657F | ATA*GGATCC*ATGTGCGCGTTCCCGTC |
|  | Rv2657R | ATA*AAGTCC*CATGCGGCACCACCGAAC |
|  | Rv2658F | ATA*GGATCC*ATGGCCGATGCGGTTAAGT |
|  | Rv2658R | ATA*AAGCTT*TTACCCGCCTAGCTGGCTC |
|  | Rv2659F | ATA*GGATCC*GTGACGCAAACCGGCAAG |
|  | Rv2659R | ATA*AAGTCC*TCACATCTCCTGGTTCTCGG |
|  | Rv2660F | ATA*GGATCC*GTGATAGCGGGCGTCGAC |
|  | Rv2660R | ATA*AAGTCC*CTAGTGAAACTGGTTCAATCCCA |
| RD14 | Rv1766F | ATA*GGATCC*GTGATTGGTGATCAAGACAGC |
|  | Rv1766R | ATA*AAGCTT*TCAAGCGAGCGCCAGG |
|  | Rv1767F | ATA*GGATCC*ATGTCGGACCAGCCACGT |
|  | Rv1767R | ATA*AAGCTT*CTAGGACGGCGTTGTGTCA |
|  | Rv1768F | ATA*GAATTC*ATGTCCTATCTCGTCGTGGTGCC |
|  | Rv1768R | ATA*AAGCTT*TCACGGCCCGGGCATCCCAT |
|  | Rv1769F | ATA*GGATCC*GTGCATGAGGTGGCTGCTCG |
|  | Rv1769R | ATA*GAATTC*TTAGAGGAAGGTGCGCCCTT |
|  | Rv1770F | ATA*GGATCC*ATGGACGAGGCCCACCCGG |
|  | Rv1770R | ATA*AAGCTT*TCATCGGGCCAGCTCCCTGA |
|  | Rv1771F | ATA*GAATTC*ATGAGCCCGATATGGAGTAATTG |
|  | Rv1771R | ATA*AAGCTT*TCAGGGACCGAGAACGCG |
|  | Rv1772F | ATA*GGATCC*TTGGGTTCAACAGGAGGTAGC |
|  | Rv1772R | ATA*AAGCTT*TTACGCCGCCGCATGCG |
|  | Rv1773F | ATA*GAATTC*GTGCCGCCTACGGAAGGAAAG |
|  | Rv1773R | ATA*AAGCTT*TCAGCGAGTCCAGTCCGGC |
| RD15 | Rv1963F | ATA*GGATCC*ATGGCATCCGTCGCCCAAC |
|  | Rv1963R | ATA*GAATTC*TATGCGCGCAAGAGGTTACC |
|  | Rv1964F | ATA*GGATCC*ATGGTAATCGTGGCCGACAAG |
|  | Rv1964R | ATA*AAGCTT*TCAGGACACCATGAATGGGATG |
|  | Rv1965F | ATA*GGATCC*ATGACGGCAGCGAAAGCCCT |
|  | Rv1965R | ATA*AAGCTT*CTAGGTCGCCAGGTGAAAGTTG |
|  | Rv1966F | ATA*GGATCC*ATGAGACGCGGGCCGGGTC |
|  | Rv1966R | ATA*AAGCTT*TCATGGCTGCTCCCCCGC |
|  | Rv1967F | ATA*GGATCC*ATGAGGGAGAACCTGGGGGG |
|  | Rv1967R | ATA*AAGCTT*TCATTTCGGCGCGCACCG |
|  | Rv1968F | ATA*GGATCC*ATGAAATCCTTCGCCGAACGC |
|  | Rv1968R | ATA*AAGCTT*TCACGGTTGGTCTCCCGGC |
|  | Rv1969F | ATA*GGATCC*GTGACAACGAAACTCAGACGTGCC |
|  | Rv1969R | ATA*AAGCTT*TCACGAGCCACCCCCCGG |
|  | Rv1970F | ATA*GGATCC*GTGAGGATCGGCCTGACCCTG |
|  | Rv1970R | ATA*AAGCTT*CTAGGGCCCCTGATCCCAGC |
|  | Rv1971F | ATA*GGATCC*ATGCTGCATCTACCGCGCCG |
|  | Rv1971R | ATA*AAGCTT*TCAGCTGCCCGGCGGCA |
|  | Rv1972F | ATA*GGATCC*ATGTCGGTAGCAGTGGATTCCG |
|  | Rv1972R | ATA*AAGCTT*TCACGGCACGAACCCGACG |
|  | Rv1973F | ATA*GGATCC*GTGAGCTGGTCGCGGGTG |
|  | Rv1973R | ATA*AAGCTT*TCAGATCGGTTCGAATTGCG |
|  | Rv1974F | ATA*GGATCC*GTGCAGCGCCAATCATTGAT |
|  | Rv1974R | ATA*AAGCTT*TCAGCCCGAGCGCCGAT |
|  | Rv1975F | ATA*GAATTC*ATGTCGCGTCGAGCATCGG |
|  | Rv1975R | ATA*AAGCTT*CTACTGCGGCGGCATTGC |
|  | Rv1976F | ATA*GGATCC*GTGCGGTGGATTGTCGACGG |
|  | Rv1976R | ATA*AAGCTT*TCACTGCGTGCGGCGGG |
|  | Rv1977F | ATA*GGATCC*ATGTCTCAGACACCCGCTACAACC |
|  | Rv1977R | ATA*AAGCTT*TCACTTCGAGGGCTGCCG |
| RD16 | Rv3400F | ATA*GGATCC*ATGGCGAACTGGTATCGC |
|  | Rv3400R | ATA*AAGCTT*CTACAGCAGCTCGGCGAGAT |
|  | Rv3402F | ATA*GGATCC*ATGAAGATCCGAACGTTATCCGGC |
|  | Rv3402R | ATA*AAGCTT*TCATTCACCGCGCACCTCCG |
|  | Rv3403F | ATA*GGATCC*ATGTTAGCCTTCCCTTATTTGATG |
|  | Rv3403R | ATA*AAGCTT*TCAGCATGGGGCCAGTGC |
|  | Rv3404F | ATA*GGATCC*GTGACGATATTGATCCTGACCG |
|  | Rv3404R | ATA*AAGCTT*TTAGGCTTCCGCGGGCTT |
|  | Rv3405F | ATA*GAATTC*ATGACTACGCGTCCGGCAAC |
|  | Rv3405R | ATA*AAGCTT*TCAGTGCGGTTCCAGGATC |

Restriction enzyme sites are in italics and underlined.

**Table S2** The coincidence rates between identified antigens and ESAT6/CFP10, TB-DOT in TB diagnosis

|  |  | TB-DOT | | ESAT6/CFP10 | | Total |  |  | TB-DOT | | ESAT6/CFP10 | | Total |
| --- | --- | --- | --- | --- | --- | --- | --- | --- | --- | --- | --- | --- | --- |
|  |  | + - | | + - | |  |  |  | + - | | + - | |  |
|  | + | 24 | 9 | 22 | 11 | 33 |  | + | 7 | 4 | 7 | 4 | 11 |
| Rv0222^a^ | _ | 3 | 2 | 3 | 2 | 5 | Rv0222^b^ | _ | 3 | 2 | 2 | 3 | 5 |
|  | Total | 27 | 11 | 25 | 13 | 38 |  | Total | 10 | 6 | 9 | 7 | 16 |
|  | + | 25 | 8 | 23 | 10 | 33 |  | + | 7 | 7 | 9 | 5 | 14 |
| Rv3403c^a^ | _ | 2 | 3 | 2 | 3 | 5 | Rv3403c^c^ | _ | 5 | 1 | 3 | 3 | 6 |
|  | Total | 27 | 11 | 25 | 13 | 38 |  | Total | 12 | 8 | 12 | 8 | 20 |

a: in PTB-SP diagnosis; b: in EPTB diagnosis; c: in PTB-SN diagnosis

**Table S3** B- and T-cell epitopes of Rv0222 and Rv3403c

| **antigens** | B cell epitopes | | CD4^+^T cell epitopes | |
| --- | --- | --- | --- | --- |
|  | Sequence | Position | Sequence | Position |
| Rv0222 | LGFTERPPTKPLI | 91-103 | RILIITINRPKAKNA | 21-35 |
|  | SRGWSPDTMF | 219-228 | GGTELALAADLIVAA | 113-127 |
|  |  |  | LGLVNVLAEPGTALD | 178-192 |
| Rv3403c | EQNKQRWLAFF | 96-106 | MLAFPYLMTMITPPT | 1-15 |
|  | NGLCRPNRTNRG | 460-471 | ACSMTLLEMADALLS | 26-40 |
|  |  |  | ADLYDAVAALFGQAL | 340-354 |
|  |  |  | FTKLVRRAGRDCRQA | 373-387 |
|  |  |  | ADGTLSLLAGEVLRV | 393-407 |

The shared epitopes of CD4^+^ and CD8^+^T cell are underlined.

**Supplementary figure legends:**

**Fig. S1 Classification of the antigenic proteins identified according to their annotations.** (A) Classification of 68 purified proteins. (B) Classification of 29 proteins of the initial screening.

**Fig. S2 Levels of antibody responses among different groups of EPTB patients, PTB patients and LTBI.** Each point represents one serum sample. The horizontal lines on each group indicate the median value. P-values of absorbance differences between each two groups were shown above the plots determined with one-way ANOVA Newman-Keuls test. *, *p*< 0.05.

**Fig. S3 Levels of antibody responses among different age groups of PTB patients and EPTB patients.** Each point represents one serum sample. The horizontal lines on each group indicate the median value.

**Fig. S4 B cell epitopes prediction using DNAstar software.(A**) B cell epitopes prediction of Rv0222; (B) B cell epitopes prediction of Rv3403c.
