# Supplementary material for: Identification of new diagnostic biomarkers for Mycobacterium tuberculosis and the potential application in the serodiagnosis of human tuberculosis
Source: Microb Biotechnol. 2018 Jun 27;11(5):893–904. doi: 10.1111/1751-7915.13291 (PMC6116745; doi:10.1111/1751-7915.13291)
Supplement: Supplementary file 6 — Data S1. Materials and methods. [file MBT2-11-893-s006.doc]

**Materials and methods**

**Cloning, expression and purification of RD antigens**

The primer pairs specific to of *M.tb* H37Rv genome (GenBank accession no:AL123456.3) were designed with the addition of restrictive enzyme sites and three protective nucleic acids at 5’ terminals (Table S1) and commercially synthesized by the company of Sangon Biotech (Shanghai) Co., Ltd, China. *M.tb* H37Rv strain was kindly offered by Prof. Li Chuanyou from Beijing Tuberculosis and Thoracic Tumor Research Institute, Beijing, China. The DNA templates were extracted from the of fresh *M.tb* culture after being inactivated at 90 °C for 30 min. The PCR reaction mixture (50μl) included the template (100ng/μl) 2μL, the forward and reverse primers (10mM) each 1μL, PrimerSTAR HS DNA polymerase(2.5U/μL) 0.5μL (TaKaRa, Dalian, China), PrimerSTAR buffer 10μL (TaKaRa, Dalian, China), dNTP mixture (2.5mM) 4μL, ddH_2_O 31.5μL (TaKaRa, Dalian, China). The reaction was programed as 95℃5min, 95℃1min, 58℃ 30 sec(varied slightly with the primers), 72℃ 1 min, 35 cycles, and final extension at 72℃ 5min. The PCR products and vector pET32a with six His-tags were restrictively digested, purified and ligated with T4 DNA Ligase (TaKaRa, Dalian, China) at 16℃ overnight. The recombinant plasmids were transformed into competent DH5α and BL21 (DE3) (Transgen, Beijing, China).

After DNA sequencing, the transformants of BL21 containing recombinant pET32a were inoculated into Luria-Bertani (LB) medium containing 50 µg/ml ampicillin (AMP)(Sigma, Shanghai, China). The cultures were grown in a shaker (Suzhou Peiying Equipment Co., Ltd. ) at 37°C for 3 h. And 0.8 mM isopropyl β-D-thiogalactoside (IPTG) (Sigma, Shanghai, China) was added to the cultures, continuing to grow for another 3 h in the shaker. The cells were collected after centrifugation, resuspended with binding buffer (0.5M NaCl, 0.02 M Tris-HCl, 0.005 M imidazole, pH 7.9), and lysed at 4-6°C by Low Temperture Ultra-high Pressure Continuous Flow Homogenizer(Guangzhou Juneng Biology＆Technology Co., LTD).

The lysate was centrifuged at 15400g for 20 min at 4°C and the supernatant and precipitate were collected separately. The soluble protein in the supernatant was purified by using nickel-nitrilotriacetic acid (Ni-NTA) resin affinity chromatography (GE healthcare, ShangHai, China), while the precipitate containing inclusion bodies was lysed with buffer A (0.05 M Tris-HCl, 0.005 M EDTA, 0.05 M NaCl, 0.005 M DL-Dithiothreito, 5% glycerin), then 20% PEG4000 (final concentration 0.2%) and oxidized (0.05 M) and reduced glutathione (0.05 M) (each final concentration 1 mM) were added into the lysate to purify proteins. The purified proteins were further concentrated through ultrafiltration. Their molecular weights and purities were checked by SDS-PAGE and Coomassie staining. The concentration of proteins was measured with BCA protein assay kit (Beijing CellChiop Biotechnology Co., Ltd ), and then stored at -80°C until use.
